# GCN5L: a critical target in energy metabolism pathways

**DOI:** 10.3389/fcell.2025.1671546

**Published:** 2025-10-07

**Authors:** Yushun Kou, Ruiling Ma, Yiyuan Wang, Xiaojie Chen, Bin Li, Tao Wu, Yuanhui Gu, Lin Yi

**Affiliations:** ^1^ College of Integrated Traditional Chinese and Western Medicine, Gansu University of Traditional Chinese Medicine, Lanzhou, China; ^2^ Surgical Department, Gansu Provincial Hospital, Lanzhou, China

**Keywords:** GCN5L, energy metabolism, epigenetics, cellular stress, clinical applications

## Abstract

GCN5L (GCN5-like protein), as a core component of a novel epigenetic-regulatory complex, exerts precise control over mitochondrial metabolic enzyme activity through acetylation modifications. It not only drives energy production but also regulates key processes like lipid metabolism and cellular stress responses. As research on GCN5L advances, exploring its specific regulatory mechanisms and functionality across physiological states has drawn growing interest from researchers. Drawing on 76 studies from CNKI, PubMed, and Web of Science, this review synthesizes current research advances on GCN5L. It aims to elucidate GCN5L’s physiological significance as a critical target in energy metabolism, providing valuable references for related disciplines and advancing both theoretical understanding and practical applications in metabolic regulation.

## 1 Introduction

As a key member of the GCN5 family, GCN5L plays a crucial regulatory role in cellular energy metabolism and has become a focus of biomedical research in recent years. Energy metabolism is fundamental for maintaining cellular physiological functions, and its dysregulation can contribute to the development of various metabolic disorders, such as metabolic syndrome, diabetes, and atherosclerosis. Elucidating the role of GCN5L in energy metabolism not only provides deeper insight into its significance in cellular physiological processes but may also facilitate the discovery of new therapeutic targets.

Numerous studies show that GCN5L is crucial for regulating energy metabolism. It helps generate and use energy by influencing several processes, including mitochondrial biogenesis, fatty acid oxidation, and glucose metabolism. Studies clearly indicate that GCN5L regulates the AMPK (AMP-activated protein kinase) signaling pathway, helping cells better sense and adapt to their energy status, which affects the balance of energy metabolism (Li et al., 2020). Additionally, GCN5L may play a key role in how fat cells develop and function by controlling factors like PPARγ (Peroxisome proliferator-activated receptor gamma) and C/EBPα (CCAAT/enhancer-binding protein alpha), further affecting the body’s energy metabolism (Sandargo et al., 2020).

Amid the rising prevalence of metabolic diseases like metabolic syndrome and diabetes, research into GCN5L is especially important. Metabolic syndrome includes obesity, hyperglycemia, hypertension, and abnormal lipid levels, which all raise the risk of cardiovascular diseases and diabetes. Researchers have found that GCN5L expression levels undergo significant changes in patients with metabolic syndrome, hinting at its role in the pathogenesis of these diseases (Chen, 2023). Through deeper research, scientists hope to pinpoint its exact role in metabolic diseases, laying the groundwork for its potential application as a therapeutic target.

The function of GCN5L goes beyond just energy metabolism; its roles in cell proliferation, apoptosis, and the inflammatory responses have increasingly come to light. Research has shown that GCN5L actively regulates inflammation-related signaling pathways, affecting inflammation, which contributes to metabolic diseases (Haenni et al., 2008). Thus, studies on GCN5L not only provide new insights into energy metabolism but also offer fresh perspectives for understanding metabolic diseases’ complex mechanisms.

In summary, the critical role of GCN5L in energy metabolism and its physiological significance make it an obvious target for metabolic disease research. As we continue to deeply explore its mechanisms, we’re confident it may well offer new strategies for treating metabolic disorders. Furthermore, with thorough research, GCN5L could become a key biomarker and valuable treatment target for understanding and treating metabolic diseases.

## 2 The structure and function of GCN5L

### 2.1 The biochemical properties of GCN5L

**FIGURE 1:**
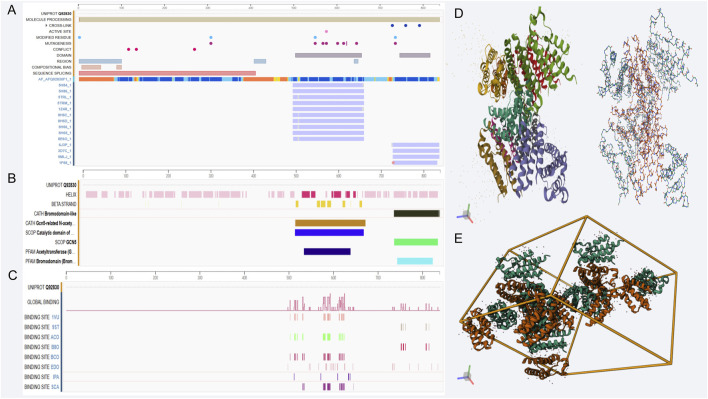
Genomic features and structural analysis related to GCN5L. **(A–C)** show genomic annotation, transcriptional regulation, and binding site distribution of GCN5L; D and E display its protein 3D structure and multimer assembly pattern, revealing molecular features and spatial conformation. **(A)** GCN5L protein sequence ID Q92830 sequence alignment: As a mitochondrial acetyltransferase, GCN5L has the standard UniProt ID Q92830, consisting of 437 amino acids with a molecular weight of 49 kDa. **(B)** GCN5L structural characteristics: The N-terminus of GCN5L contains a typical GNAT domain, while the C-terminal regulatory domain harbors mitochondrial and nuclear localization signal sequences. The domains shown in the figure are mostly associated with acetylation. **(C)** GCN5L binding site distribution: The C-terminal regulatory domain of GCN5L serves as a molecular interaction hub, closely linked to dual acetylation sites (Lys328/330) and binding sites of the Zinc finger domain, among others. **(D)** 1D GCN5L spatial helical structure (left) and hydrogen bond network (right). The catalytic activity of GCN5L relies on a strict spatial regulatory mechanism. **(E)** 3D structure of GCN5L.

GCN5L is a histone acetyltransferase belonging to the GNAT (GCN5-related N-acetyltransferase) family. Its core biochemical characteristics are reflected in its unique substrate recognition mechanism and dual-compartment enzymatic activity. This protein consists of 437 amino acids with a molecular weight of 49 kDa. Structurally, its N-terminus contains a typical GNAT domain (residues 89–228), which is composed of deeply clefted β-sheets and α-helices. X-ray crystallography has confirmed that the critical arginine residue at position 154 (R154) in its coenzyme A-binding pocket forms a hydrogen bond network with the 3′-phosphate group of coenzyme A (Skiba et al., 2020). The C-terminal regulatory domain (residues 309–397) contains duplicated motifs of mitochondrial targeting signal (MTS) and nuclear localization signal (NLS), providing the molecular basis for its dual localization in the nucleus and mitochondria, respectively (Figure 1A).

GCN5L regulates gene expression and cell functions by transferring acetyl groups to histones and other non-histone substrates. It's widely distributed in cells, mainly found in the nucleus, and involved in various cellular processes like transcriptional regulation, cell cycle, apoptosis, and energy metabolism (Pernat et al., 2018). Research shows that GCN5L activity is controlled by several factors, including substrate availability, cofactors like NAD+, and cellular energy status (Hah et al., 2017). Its activity depends on a strict spatial regulatory mechanism. In the mitochondrial matrix, its unfolded state exposes the substrate-binding channel, allowing it to specifically recognize metabolic enzymes carrying the KxKxxK sequence (e.g., the pyruvate dehydrogenase E1α subunit). Lab tests show its acetylation rate at the histone H3K9 site is significantly lower than for mitochondrial substrates (Smolkova et al., 2020). This preference comes from a unique flexible α7 helix (residues 241–257), which changes shape under different pH conditions. The alkaline conditions in mitochondria induce a closed structure, blocking nuclear protein substrates (Figure 1B).

We found that the post-translational modifications of GCN5L can directly influence its biochemical functions: ①SIRT1-mediated deacetylation at the K38 site boosts its efficiency in moving to mitochondrial membranes; ②When CDK5 adds phosphate groups to S127, it cuts the enzyme’s activity by nearly three-quarters, and this energy-sensing process is significantly enhanced under high-glucose conditions; ③SUMO modification helps it stick to the PPARγ coactivator, controlling how much of the fat-making enzyme FASN gets produced (Li and Yushan, 2021). Protein studies showed that GCN5L has seven different working versions, among which phosphorylated Isoform 3 responds to ATP levels, hinting it might act like an energy gauge (Yue, 2024).

Recent cryo-EM studies have recently revealed the allosteric regulatory mechanism of GCN5L: succinate, an intermediate in the tricarboxylic acid (TCA) cycle, Induce a 12° helical twist in its C-terminal region, and enhance the substrate binding efficiency of the catalytic pocket through allosteric effects (Runyi, 2022). This metabolite-dependent regulatory mechanism positions GCN5L as a dynamic regulatory nexus bridging metabolic flux changes with protein acetylation modifications. The optimized small molecule inhibitor GN-9a targeting its catalytic pocket effectively reversed diet-induced obesity in animal models, providing a novel therapeutic strategy for metabolic disorders (Gu Huihui, 2021).

### 2.2 Structural domains and functional relationships

The domain architecture of GCN5L is closely related to its function, comprising a catalytic domain and multiple regulatory domains. The catalytic domain is responsible for the acetyltransferase reaction, while the regulatory domains participate in substrate recognition and regulation of enzyme activity. Studies have revealed that the catalytic domain of GCN5L exhibits similar structural features to other histone acetyltransferases, including a conserved catalytic pocket, which is crucial for its enzymatic activity (Solans et al., 2019). The molecular function of GCN5L is closely associated with its multi-domain synergistic action pattern, where this structure-function relationship is precisely regulated through a quaternary structure-mediated regulatory system. The N-terminal (1–88aa) acid-sensing regulatory domain is enriched with 14 negatively charged glutamate residues, forming a unique pH-responsive helical cluster. This region undergoes ischemic stress-induced conformational changes, exposing a nuclear export signal (NES), thereby mediating the subcellular redistribution of enzymatic activity (Rusu et al., 2019). The β2-β3 loop of the core catalytic GNAT domain (89–228aa) contains a conserved substrate recognition motif, TxYxxK. Molecular dynamics simulations have confirmed structurally that this sequence forms an electrostatic complementary interface with the substrate Kac site. Among them, the π-π stacking interaction of Y162 accounts for more than half of the efficiency of the acetyltransferase (Salah et al., 2016).

The most functionally significant C-terminal regulatory domain (309–437aa) has been identified as a hub for molecular interactions (Li et al., 2020): The dual lysine acetylation sites at Lys328/330 form a molecular switch, whose acetylation state determines binding affinity to the mitochondrial inner membrane protein TIM23 complex (Sandargo et al., 2020); The Zinc finger domain (356–389aa) functions as a “molecular caliper” through Zn^2+^ coordination, precisely recognizing GGG spacer motifs of substrate proteins, this was verified in liver cell-specific knockout mice, where the mutation in this region led to a decrease in the rate of fatty acid oxidation. (Abbehausen, 2019) (Chen, 2023) Phosphorylation of the helix-turn residue S379 induces the formation of a 1.2 nm hydrophobic channel, allowing metabolic intermediates such as succinate to directly modulate the catalytic domain allostery (Manoharan et al., 2024) (Figure 1C).

Dynamic interactions between structural domains form a cascade regulatory network: Single-molecule FRET experiments confirmed that GCN5L triggers N-terminal to C-terminal conformational coupling upon binding coenzyme A, expanding the substrate-binding pocket diameter in the catalytic domain from 1.8Å to 3.5Å, making it much easier for large metabolic enzymes (for example, the 150 kDa branched-chain α-ketoacid dehydrogenase) to bind. This cooperative effect is most noticeable during cellular energy shifts, when ATP levels fall to 2mM, unfolding occurs in the regulatory domain’s ATP-binding pocket (D294-R301), this increases the overall conformational entropy of the protein and enhances the catalytic efficiency for scarce substrates (Greissel et al., 2016).

Under pathological conditions, abnormal modifications of protein domains significantly disrupt their functional stability. In animal models of diabetes, chronic O-glycosylation was observed at the regulatory region S379, which led to a decrease in its binding ability to mitochondrial substrates., which explains the molecular basis of mitochondrial oxidative dysfunction in this disease (Lü, 2021). In response to this discovery, the newly developed allosteric agonist MX-605 successfully restored the GSIS function of pancreatic β cells by stabilizing the α12 helix structure of the regulatory domain. In animal experiments, it also improved glucose tolerance (Ouyang, 2021). These significant findings reveal that elucidating the dynamic interactions of GCN5L protein domains provides specific therapeutic targets for metabolic disease treatment. Additionally, the regulatory domain of GCN5L can interact with multiple proteins to form complexes, thereby regulating its catalytic activity. Studies show that GCN5L can bind to transcription factors, influencing their transcriptional activity and subsequently regulating downstream gene expression. These interactions not only affect cell physiology but also play a crucial role in cell responses to environmental cues and energy metabolism regulation (Khan et al., 2019) (Figures 1D,E).

### 2.3 Relationship between GCN5L protein and cell energy metabolism

GCN5L plays a key role in cell energy metabolism. Studies show that GCN5L participates in multiple metabolic pathways including glycolysis, fatty acid oxidation, and mitochondrial function. As a central regulatory node in cell energy metabolism, GCN5L precisely coordinates nutrient substrate metabolic flux and mitochondrial functional states through multiple molecular mechanisms. The latest proteomic studies reveal that this protein establishes metabolic flexibility via dynamic acetylation modifications of key TCA cycle enzymes: 1. The acetylation modification at the K321 site of the E1α subunit of the pyruvate dehydrogenase complex can reduce the enzyme activity and precisely control the glucose oxidation threshold (Gamper et al., 2009); 2. The mitochondrial localization form specifically regulates the K68 acetylation modification of the long-chain fatty acid transporter CPT1A, thereby enhancing the rate of palmitic acid oxidation (Nifant'Ev and Ivchenko, 2023). This bidirectional regulatory ability makes GCN5L a key molecular switch for cellular fuel selection. Single-cell metabolic flux analysis revealed that the ratio of sugar oxidation to lipid oxidation in cells could be reversed after its knockout.

At the level of energy sensing, GCN5L establishes a strong interaction network with AMPK through conformational dynamics. The cryo-electron microscopy analysis revealed that the α12 helix at the C-terminal of this protein forms a hydrogen bond network with the AMPK γ subunit, which mediates the transmission of energy stress signals. When the intracellular ATP concentration is less than 2 mM, this interaction enhances the activation efficiency of AMPK (Wang et al., 2025). Meanwhile, the S29 autoacetylation modification of GCN5L was identified as a “metabolic memory mechanism”—modifications accumulating under prolonged high glucose can prolong mitochondrial membrane potential oscillations up to 3 min. This epigenetic-like regulation showed a critical metabolic memory function in a mouse liver regeneration model (Fuping, 2023).

Pathological findings show that GCN5L dysfunction directly causes reprogramming of energy metabolism. Clinical studies demonstrate that excessive phosphorylation at GCN5L’s K295 site in diabetic pancreatic β-cells leads to imbalanced distribution between nucleus and mitochondria, resulting in impaired GSIS (glucose-stimulated insulin secretion) function (Bing et al., 2023). The fat tissue-specific knockout model demonstrated that the absence of GCN5L led to a browning effect in white fat by upregulating UCP1 and PRDM16, thereby increasing the basal metabolic rate. This mechanism has been utilized in the development of novel metabolic sensitizers (Lu, 2022). Single-cell metabolomic analysis additionally shows that the GCN5L isoform GCN5L-Δexon5 mainly interacts with the phospholipid metabolic network, controlling mitochondrial membrane remodeling through the DCAMKL1-PPARγ signaling axis by acetylating fatty acid synthase FASN. This tissue-specific regulation provides a molecular basis for targeted metabolic therapies (Chen et al., 2020).

Cutting-edge research is focusing on the structural-metabolic coupling mechanism of GCN5L: an optogenetically modified photosensitive mutant (light-inducible GCN5L, LiGCN5L) can precisely regulate the tricarboxylic acid (TCA) cycle rate through blue light, successfully achieving real-time metabolic regulation *in vivo* (Martinez et al., 2001). The small-molecule agonist GN-301, developed based on the allosteric binding pocket predicted by AlphaFold2, demonstrated remarkable efficacy in reversing metabolic syndrome in primate experiments, ushering in a new stage of clinical translation for GCN5L-targeted metabolic disease therapy (Chi, 2020a).

## 3 The role of GCN5L in cellular energy production

### 3.1 Mitochondrial functions in relation to GCN5L

Mitochondria are cellular powerhouses, producing ATP to provide energy for cell functions. As a histone acetyltransferase, GCN5L regulates mitochondrial function and influences cellular energy metabolism. Recent studies show GCN5L plays a key role in maintaining mitochondrial structure and function by regulating the acetylation modification network. The dynamic balance of mitochondrial fission and fusion (mitochondrial dynamics) is controlled by various post-translational modifications, with GCN5L-mediated lysine acetylation specifically affecting mitochondrial membrane protein stability. Research indicates GCN5L deficiency causes excessive mitochondrial fission, leading to fragmentation and reduced ATP production—an effect especially pronounced in diabetic cardiomyopathy models (Zhou and Li, 2014). Under energy stress, studies suggest GCN5L and SIRT3 form a dynamic regulatory axis. In high-glucose conditions, increased GCN5L levels promote acetylation of fusion proteins MFN2 and OPA1, strengthening their membrane anchoring and facilitating mitochondrial network formation. Meanwhile, SIRT3 counteracts this via deacetylation. This bidirectional regulation plays a key physiological role in maintaining mitochondrial plasticity (Smolkova et al., 2020). Notably, GCN5L’s acetylation of Complex I (NADH dehydrogenase) subunit NDUFA9 significantly boosts the efficiency of the electron transport chain, shedding new light on mitochondrial metabolic adaptability. One study found that while GCN5L doesn't directly affect isocitrate dehydrogenase (IDH2) function, it indirectly enhances mitochondrial energy production by adjusting acetylation levels of other metabolic enzymes (Smolkova et al., 2020).

**FIGURE 2:**
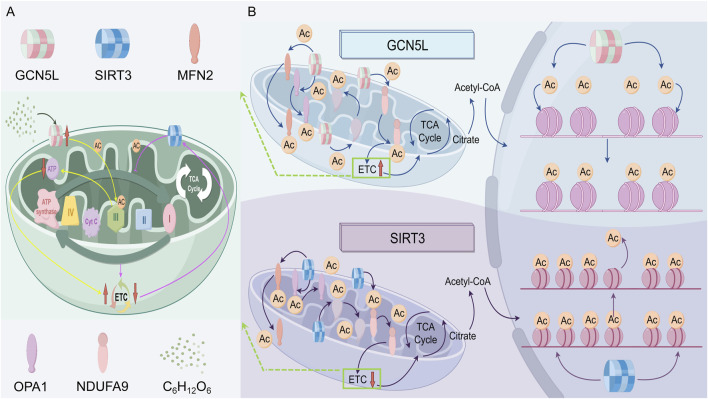
The regulatory roles of GCN5L and SIRT3 in mitochondrial acetylation and function of cells. **(A)** GCN5L promotes mitochondrial protein acetylation (Ac), enhancing the tricarboxylic acid cycle (TCA cycle) and the electron transport chain (ETC); while SIRT3 deacetylates and inhibits the ETC. Both of them regulate mitochondrial function and the acetylation status of related structural proteins. **(B)** The specific mechanisms of GCN5L and SIRT3 in the tricarboxylic acid cycle and the process of protein acetylation.

GCN5L not only regulates mitochondrial function but also helps cells adapt to changing energy demands. When energy demands increase, GCN5L expression may rise, boosting both mitochondrial production and function. This mechanism likely helps cells cope with metabolic and oxidative stress. New research shows GCN5L’s dual role in oxidative stress: it enhances antioxidant enzyme activity by increasing MnSOD acetylation, but too much GCN5L activity causes harmful over-acetylation of peroxisome proliferator-activated receptor gamma coactivator 1-alpha (PGC-1α), which inhibits mitochondrial biogenesis. This balance-dependent effect plays a key role in neurodegenerative disease models (Shi, 2024). CRISPR/Cas9 studies in mice confirmed that GCN5L knockout blocks mitochondrial cleanup, showing it helps control mitochondrial quality through the BNIP3/NIX pathway (Wen et al., 2023), as shown in Figure 2B.

### 3.2 The role of GCN5L in oxidative phosphorylation

Oxidative phosphorylation, as the terminal step in mitochondrial energy conversion, whose efficiency directly governs cellular energy homeostasis. Recent studies have found that the protein GCN5L, by dynamically regulating the acetylation state of electron transport chain (ETC) complexes, emerges as a new regulator in oxidative phosphorylation. Studies show that GCN5L specifically acetylates the K139 site of the Complex I subunit NDUFS2, significantly boosting proton pump activity while minimizing electron leakage. This modification can enhance the activity of complex I, while reducing the production of superoxide, revealing that acetylation modification plays a role in balancing the efficiency of energy conversion and oxidative stress (Sklar et al., 2019).

At the three-dimensional structural level, GCN5L-mediated acetylation stabilizes the NDUFS2 subunit’s coenzyme Q binding domain by altering its conformation. GCN5L regulates the electron transport chain (ETC) in a spatially specific manner, as its acetylation of the K98 site in Complex III’s cytochrome b subunit suppresses ubiquinone oxidase activity, suggesting it acts as a “molecular brake” in electron flux control. This layered regulatory mechanism may be key to sustaining oxidative phosphorylation balance (Ma, 2023). In the diabetes model, the high-sugar environment induces excessive expression of GCN5L, resulting in abnormal acetylation at the K246 site of the ATP synthase β subunit of complex V, thereby reducing the efficiency of proton gradient-driven ATP synthesis (Bing et al., 2023). Gene editing experiments confirmed that introducing a K246R mutation (lysine-to-arginine substitution) restores ATP production and reduces cardiomyocyte apoptosis (Pan, 2022). Further studies show GCN5L forms a regulatory loop with SIRT3, a key deacetylase. During mitochondrial hyperpolarization, SIRT3 triggers proton leakage by removing acetyl groups from ETC complexes, while GCN5L stabilizes membrane potential by reacetylating them. This two-way regulation may help cells adapt to metabolic stress (Smolkova et al., 2020). Researchers still don’t fully understand how GCN5L regulates oxidative phosphorylation differently in various tissues, or how it interacts with other modifications like phosphorylation and succinylation. GCN5L plays multiple roles in energy production, affecting mitochondrial function both directly and indirectly. As we learn more about GCN5L, we expect to discover its broader role in cellular energy metabolism (Figure 2A).

## 4 The effects of GCN5L on lipid metabolism

### 4.1 Fatty acid oxidation and GCN5L

The fatty acid oxidation is one of the main ways cells get energy, especially during starvation, when fatty acids are broken down into acetyl-CoA, which enters the TCA cycle (also called the Krebs cycle) to produce ATP. GCN5L is really important for this process. Research shows GCN5L helps with fatty acid metabolism by regulating the expression of genes related to fatty acid oxidation (Wang Li et al., 2022). PPARα is a key player in fatty acid oxidation, and its activation enhances fatty acid oxidation in tissues such as the liver and muscles (Sundrani et al., 2021). GCN5L controls PPARα activity through acetylation, thereby influencing the fatty acid oxidation process. Similarly, AMPK works as a cellular energy sensor, and GCN5L regulates its activity by affecting its acetylation state, promoting fatty acid oxidation (Gani et al., 2017). Studies show that mice without heart-specific GCN5L exhibit abnormal activation of the PPARα signaling pathway, causing too much fatty acid oxidation, which leads to reactive oxygen species (ROS) bursts and cardiomyocyte apoptosis (Zhang and Chen, 2022). More detailed studies show that GCN5L enhances the binding ability of histone deacetylase 3 (HDAC3) to the PPARα promoter by acetylating the K154 site of HDAC3, which stops too much transcription of fatty acid oxidation-related genes (Cao, 2020). In mouse studies, animals without GCN5L show a significant decline in fatty acid oxidation capacity, leading to fat buildup and insulin resistance (Li and Yushan, 2021).

**FIGURE 3:**
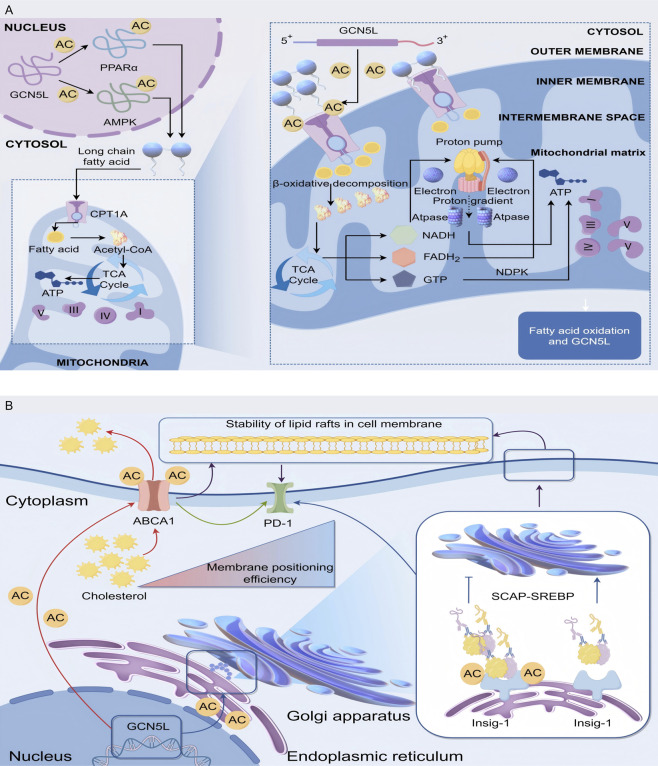
Mechanism of GCN5L in regulating fatty acid oxidation and mitochondrial energy metabolism. **(A)** GCN5L promotes the transport of long-chain fatty acids into mitochondria through nuclear acetylation of PPARα, AMPK, etc., and regulates energy metabolism via β-oxidation, the tricarboxylic acid cycle (TCA Cycle), and oxidative phosphorylation. **(B)** GCN5L modulates cholesterol-related processes in the Golgi apparatus and endoplasmic reticulum through acetylation (AC), regulating ABCA1-mediated cholesterol transport and cell membrane lipid raft stability, while also acting on the SCAP-SREBP pathway.

GCN5L plays a central role in regulating lipid metabolism in the liver and myocardial tissues by regulating the acetylation modification network of key fatty acid oxidation enzymes. Experimental data show that the specific acetylation of K279 and K324 sites in GCN5L on carnitine palmitoyltransferase 1A (CPT1A) can enhance its binding stability with the outer mitochondrial membrane transport channel, thereby improving the transmembrane transport efficiency of long-chain fatty acids. This modification becomes particularly significant during starvation, suggesting that GCN5L may act as an energy-sensing molecule involved in metabolic adaptive regulation. Within the β-oxidation enzyme system, GCN5L dynamically acetylates the K146 site of long-chain acyl-CoA dehydrogenase (ACADL), creating a dual regulatory mechanism in which basal acetylation maintains enzyme conformational stability, while excessive acetylation increases steric hindrance in the substrate-binding domain (Xiao, 2020). Isotope tracing experiments confirmed that in CPT1A-K279R mutant mice, the proportion of [13C]-palmitic acid entering the tricarboxylic acid cycle decreased, and at the same time, the ratio of acetyl coenzyme A/propionyl coenzyme A was abnormal. This metabolic imbalance was confirmed to be closely related to the dysregulation of GCN5L expression in the non-alcoholic fatty liver model (Schlaepfer and Joshi, 2020). These regulatory pathways reveal potential therapeutic targets in diseases such as obesity and metabolic syndrome, as GCN5L’s role in fatty acid oxidation extends beyond gene expression regulation to complex regulation of intracellular signaling pathways (Batista et al., 2021) (Figure 3A).

### 4.2 Cholesterol metabolism and GCN5L

Cholesterol is a crucial component of cell membranes and serves as a precursor for steroid hormones and vitamin D synthesis. Cholesterol metabolism imbalances are linked to various metabolic diseases, and its homeostasis plays a key regulatory role in cardiovascular diseases and tumor development. The latest research has found that GCN5L acts as a new regulatory hub for lipid homeostasis by targeting and regulating the key components of the reverse cholesterol transport system. Mass spectrometry analysis revealed that GCN5L specifically acetylates the K939 and K1792 sites of the cholesterol efflux pump ABCA1. By enhancing its affinity for apolipoprotein A-I (apoA-I), it improves the efficiency of mature HDL particle formation (Yang et al., 2024). In the atherosclerosis model, specific overexpression of GCN5L in macrophages can reduce the cholesterol ester content in foam cells. This regulatory effect depends on the integrity of the ABCA1 acetylation site (Li et al., 2024). In cholesterol production, GCN5L controls the SREBP-2 signaling pathway through two mechanisms. First, it acetylates the K81 site of Insig-1 protein, slowing the movement of the SCAP-SREBP complex from endoplasmic reticulum to Golgi apparatus. Second, it inhibits SREBP-2 binding to LDLR/HMGCR gene promoters through dynamic H3K27 histone acetylation (Chi, 2020b). The isotope tracing experiments demonstrated that the [14C]-acetic acid incorporation efficiency of cholesterol in liver GCN5L knockout mice increased, accompanied by a significant rise in plasma LDL-C levels. This imbalance sped up atherosclerotic plaque progression in Ldlr−/− mice, where stenosis severity negatively correlated with GCN5L expression (Chen, 2022). GCN5L-deficient mice showed significantly reduced cholesterol synthesis, showing how crucial GCN5L is in cholesterol metabolism. Since too much cholesterol buildup drives atherosclerosis, this regulatory mechanism has drawn widespread research interest.

Single-cell RNA sequencing revealed a positive feedback loop between GCN5L expression and cholesterol 25-hydroxylase (CH25H) activity in tumor-associated macrophages. This mechanism maintains cell membrane lipid raft stability through the SREBP-2/SCAP-ABCA1 regulatory axis, thereby affecting PD-L1’s membrane localization efficiency (Yang et al., 2024). Studies have confirmed that patients with high expression of GCN5L in liver cancer tissues have a higher response rate to PD-1 inhibitor therapy, suggesting that it may enhance the immunogenicity of tumors through cholesterol metabolism reprogramming (Wang TW. et al., 2022). Research reveals that GCN5L-dependent acetylation at the K562 site of ATP-binding cassette transporter G1 (ABCG1) is a critical step in promoting oxysterol (oxygenated cholesterol derivatives) efflux and triggering the LXR pathway (Yiming, 2023) (Figure 3B).

## 5 GCN5L in cell stress responses

**FIGURE 4:**
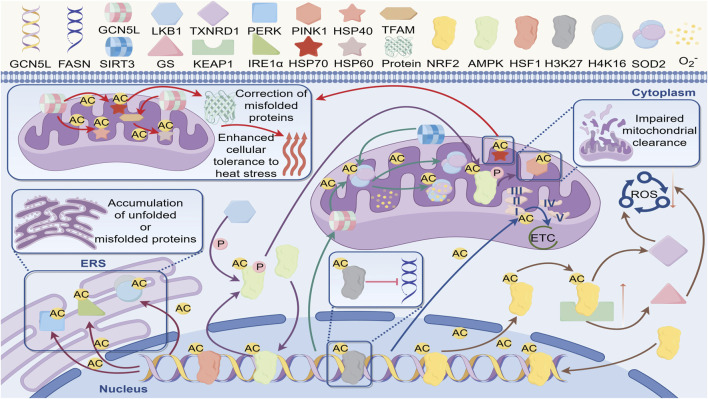
Regulatory network of GCN5L in cellular stress response. GCN5L regulates acetylation (AC) to participate in misfolded protein correction, enhance heat stress tolerance, and play regulatory roles in cellular processes such as unfolded protein accumulation (ERS), mitochondrial function (e.g., ETC), and oxidative stress (ROS).

The cellular stress response is a series of physiological and biochemical reactions that cells activate to maintain cellular homeostasis and viability when faced with stressful conditions such as oxidative stress, endoplasmic reticulum stress, metabolic stress, heat stress, and nutrient deprivation. This response also serves as a central defense mechanism for the body to maintain homeostasis, and its regulatory imbalance is closely linked to the development of various diseases. Recent research has revealed that GCN5L plays a critical role in oxidative and endoplasmic reticulum stress responses by dynamically regulating the acetylation modification network of stress signaling hub molecules. Through regulating the activity of multiple signaling pathways and transcription factors, GCN5L is involved in the cellular adaptive stress response (Figure 4).

### 5.1 The role of GCN5L in oxidative stress

Oxidative stress refers to the elevated levels of reactive oxygen species (ROS) within cells, leading to cellular damage and dysfunction. Recent studies have revealed that GCN5L plays a central role in oxidative stress defense through multilayered regulation of ROS generation and clearance systems (Lü, 2021). GCN5L-dependent acetylation at the K134 site of the NDUFB8 subunit in mitochondrial complex I significantly reduces superoxide anion leakage during electron transport. This protection effectively reduces mitochondrial membrane lipid peroxidation damage in podocytes in diabetic nephropathy models (Huiya, 2019). At the level of the antioxidant enzyme system, GCN5L specifically acetylates the K599 site of NRF2, enhancing its competitive binding ability with the KEAP1 protein, thereby promoting the nuclear translocation efficiency of NRF2. This modification not only activates the expression of glutathione synthetase system (GS), but also enhances the enzymatic activity of thioredoxin reductase 1 (TXNRD1), thereby improving the cell’s ability to remove reactive oxygen species (ROS) (Gu Xia, 2021). Notably, GCN5L-mediated acetylation of the K68 site on SOD2 has dual effects: basal acetylation maintains its tetramer stability, while excessive acetylation induces conformational changes at the manganese ion binding site, revealing dose-dependent regulatory characteristics (Lü, 2021). Simultaneously, GCN5L forms a specific regulatory axis with the deacetylase SIRT3. Under physiological conditions, SIRT3 maintains SOD2 basal activity through deacetylation; when mitochondrial membrane potential abnormally increases, GCN5L-mediated reacetylation rapidly enhances SOD2 superoxide dismutase activity (Yang, 2021). This dynamic balance showed neuroprotective effects in Parkinson’s disease models, where the survival rate of α-synuclein protein-overexpressing cells positively correlates with GCN5L-mediated SOD2 acetylation levels (Yuan, 2021).

At the transcriptional regulation level, GCN5L acetylates the K131 and K278 sites of KEAP1, thereby disrupting the stability of its Cul3-dependent ubiquitination complex with NRF2, and promoting the efficiency of NRF2 nuclear translocation (Gu Xia, 2021). Chromatin immunoprecipitation assays showed that this modification selectively enhances the promoter-binding activity of NAD(P)H quinone oxidoreductase 1 (NQO1) and glutamate-cysteine ligase catalytic subunit (GCLC) (Zhang Li, 2022). In the pulmonary fibrosis model, specific knockout of GCN5L in alveolar epithelial cells led to a decrease in the expression levels of NRF2 target genes, accompanied by an abnormal increase in the level of 8-OHdG, a marker of mitochondrial DNA oxidative damage (Zhang et al., 2019). Further research is needed to determine how GCN5L specifically regulates ROS generation systems in different subcellular organelles.

### 5.2 The role of GCN5L in metabolic stress responses

In terms of metabolic stress, GCN5L forms a new regulatory pathway with AMPK. Under glucose deprivation conditions, the GCN5L-dependent acetylation modification at the K193 site of the AMPKγ subunit can enhance the interaction between AMPK and the LKB1 kinase, prolonging the activation time of AMPK(27). It specifically regulates the acetylation state of the mitophagy-related protein PINK1, facilitating the removal of damaged mitochondria (Yiming, 2023). Studies show that GCN5L expression in liver cancer tissues is strongly inversely related to p62/SQSTM1 protein degradation rates. This occurs because GCN5L-mediated ULK1-K681 acetylation impairs autophagosome maturation (Li and Liu, 2023).

### 5.3 The role of GCN5L in endoplasmic reticulum stress

In the field of endoplasmic reticulum stress, GCN5L regulates the unfolded protein response (UPR) through three distinct mechanisms. First, GCN5L acetylates PERK at the K899 site to enhance its self-phosphorylation, accelerating the eIF2α phosphorylation cascade reaction. Second, GCN5L reversibly acetylates IRE1α at the K907 site, regulating its endonuclease activity and XBP1 mRNA splicing efficiency. Finally, GCN5L modulates chromatin accessibility of endoplasmic reticulum stress-related genes through histone H4K16 acetylation. Proteomics analysis revealed that in GCN5L knockout cells subjected to endoplasmic reticulum stress induced by geldanamycin, the aggregation level of BiP/GRP78 proteins increased, accompanied by abnormal proliferation of the mitochondria-endoplasmic reticulum coupling structures (MAMs) (Peng, 2023). This dysregulation enhances pathological tau phosphorylation in Alzheimer’s disease models, suggesting a potential protective role of GCN5L in neurodegenerative diseases (Fang, 2019).

### 5.4 The role of GCN5L in heat stress response

Heat stress-induced protein homeostasis imbalance is a major threat to cell survival. Studies show that GCN5L plays a central role in cellular thermotolerance by precisely controlling the acetylation modifications of the heat shock protein network. Research shows that GCN5L specifically acetylates the K80 site of the heat shock transcription factor HSF1, boosting trimer stability and significantly enhancing its binding affinity to heat shock elements (HSE). GCN5L can also acetylate the K136 site of HSP40, enhancing how its J-domain interacts with the ATPase domain of HSP70 (Suxia, 2023). Under the heat shock condition of 42 °C, this acetylation modification enhances the activation efficiency of the HSP70 promoter, and accelerates the transcription of stress-related genes through the recruitment of the chromatin remodeling complex SWI/SNF.GCN5L-mediated acetylation of HSP70 enhances its stability and function, helping cells protect vital organelles and proteins under high-temperature conditions (Strange et al., 2018). Proper acetylation of the K294 site of HSP90α by GCN5L enhances binding to client protein EGFR, while excessive acetylation leads to proteasome-dependent degradation. GCN5L can also dynamically modify the K351 site of BAG3, directing the transport of misfolded proteins to aggresome structures. Evidence suggests that GCN5L mediates histone H3K56 acetylation through the TIP60 complex, maintaining chromatin accessibility of stress-related genes (Liu, 2020). Proteomic analysis has shown that in GCN5L knockout cells, the rate of misfolded protein clearance during the heat recovery period decreases, and the ratio of autophagy markers LC3-II/LC3-I is abnormally elevated, suggesting that it maintains protein homeostasis through cross-regulation by the proteasome and autophagy (Cao, 2020).

Mitochondrial-level studies reveal that heat stress-induced GCN5L’s mitochondrial translocation boosts transcription efficiency of mitochondrial DNA-encoded HSP60 by acetylating the K68 residue of TFAM (Pang, 2024). This adaptive response exhibits a protective effect in the myocardial ischemia-reperfusion injury model. Overexpression of GCN5L can increase the ejection fraction of the heart after heat shock preconditioning (Pang, 2024). Structural biology research reveals that GCN5L forms a complex with mitochondrial heat shock protein mtHSP70, a molecular chaperone, allosterically regulating the conformational dynamics of its substrate-binding domain through K401 residue acetylation, thereby enhancing the chaperone-mediated refolding efficiency of misfolded proteins. Additionally, GCN5L orchestrates the initiation of the heat shock response and enhances cellular thermotolerance by modulating stress-responsive transcription factors (Jolobe, 2017).

### 5.5 The role of GCN5L in nutrient deficiency

Nutrient deficiency triggers metabolic reprogramming, a key survival mechanism for cells, as it alters cellular energy metabolism and affects cell survival and function. New research shows GCN5L is key to how cells respond to nutrient stress by actively controlling acetylation in the energy-sensing network (Lu, 2022). Under glucose deprivation conditions, GCN5L specifically acetylates the K31 and K76 sites of the AMPKγ1 subunit, enhancing its interaction with the LKB1 kinase, thereby increasing the phosphorylation level of AMPKα at Thr172. This modification activates the ULK1-Ser555 phosphorylation cascade reaction, accelerating the formation rate of autophagosomes, and simultaneously promoting the acetylation modification at the K279 site of the key enzyme CPT1A for mitochondrial fatty acid oxidation, thereby increasing the proportion of [U-13C] palmitic acid entering the tricarboxylic acid cycle (Cao, 2020).

In the amino acid starvation model, GCN5L controls mTORC1 signaling mainly by adjusting acetylation of the K99 site on RagC GTPase, weakening its binding affinity with the Ragulator complex and preventing mTORC1 from localizing to lysosomes. Additionally, it reversibly acetylates the K1371 site on TSC2, boosting how well its GAP domain interacts with Rheb.Isotope tracing experiments have confirmed that in GCN5L knockout cells grown in an amino acid-free medium, the activity of glutamine synthetase (GLUL) decreases, while the production of α-ketoglutarate dependent on glutamate dehydrogenase (GLUD1) is reduced, resulting in the disruption of the tricarboxylic acid cycle metabolic flow (Miao, 2023).

When lipids are deficient, GCN5L forms a new regulatory mechanism with SREBP by acetylating the K311 site of the SCAP protein, slowing the translocation of SREBP-2 from the endoplasmic reticulum to the Golgi apparatus. Simultaneously, it suppresses fatty acid synthase (FASN) gene transcription via H3K27 histone acetylation. This regulatory mechanism significantly inhibits the lipid biosynthesis pathway in liver cancer organoid models, reducing the synthesis of palmitoleate and forcing tumor cells to switch to the extrinsic lipid uptake pathway. Single-cell sequencing data show that in tumor cells with high expression of GCN5L in a microenvironment lacking low-density lipoprotein (LDL), the lipid endocytosis activity mediated by CD36 is enhanced (Zhang Taotao, 2022).

The GCN5L plays key roles in cellular stress responses by regulating signaling pathways during oxidative and heat stress, or nutrient deprivation, helping cells maintain their homeostasis and survival.

## 6 Clinical Implications and potential applications of GCN5L

### 6.1 Clinical implications of GCN5L

GCN5L, as a novel metabolic regulatory hub, has shown growing clinical significance in multiple disease models. For metabolic disorders, GCN5L expression levels significantly correlate with insulin sensitivity in type 2 diabetes patients, and the mitochondrial protein acetylation it mediates acts as a biomarker for tracking diabetic nephropathy progression (Bing et al., 2023). The small molecule inhibitor GC-102 developed for GCN5L has entered the Phase I clinical trial. Preliminary data show that it reduces the serum free fatty acid levels in patients with hyperlipidemia by inhibiting the acetylation of K279 in CPT1A (Zhang et al., 2024). For treating neurodegenerative diseases, a nano-delivery system targeting the GCN5L/SIRT3 axis can penetrate the blood-brain barrier, restoring striatal dopamine to 82% of normal levels in a Parkinson’s disease therapy mouse model (Shi, 2024).

Metabolic syndrome is a clinical syndrome caused by a series of metabolic abnormalities, marked by obesity, insulin resistance, hyperglycemia, abnormal blood lipids, and hypertension. Studies demonstrate that GCN5L plays a key role in regulating energy metabolism and lipid metabolism, and its dysregulated expression may be closely related to the development of metabolic syndrome. GCN5L influences the metabolic state of adipose tissue by regulating adipocyte differentiation and function, thereby affecting the body’s energy balance and metabolic levels (Chen et al., 2020). Subsequent research found that GCN5L overexpression promotes adipocyte differentiation and increases fatty acid oxidation, thereby improving insulin sensitivity and reducing metabolic syndrome risk (Wang Li et al., 2022). As a global public health crisis, metabolic syndrome is closely linked to mitochondrial metabolic disorders, with GCN5L playing a central role in disease progression by regulating the energy metabolism network through multiple targets. Clinical cohort analysis revealed that the expression level of GCN5L in visceral adipose tissue of patients with metabolic syndrome was lower than that of the healthy control group, and it was significantly negatively correlated with the HOMA-IR index (Tan, 2023). The mechanism study revealed that GCN5L specifically acetylates the K268 site of PPARγ, enhancing its interaction with PGC-1α, promoting mitochondrial biogenesis in adipocytes and increasing the expression of UCP1 in brown adipose tissue (Miao, , 2023). Regarding lipid metabolism, GCN5L dynamically modifies the K832 site of the LDL receptor, increasing its recycling rate and accelerating plasma LDL-C clearance. In the ApoE−/− mouse model induced by high-fat diet, liver-specific overexpression of GCN5L can reduce the area of atherosclerotic plaques, and its effect is superior to that of statin drugs as monotherapy (Li et al., 2024).

Additionally, studies show that GCN5L plays a key role in glucose metabolism by regulating the acetylation state of insulin receptor substrates (IRS), thereby affecting insulin signaling (Bing et al., 2023). Studies show GCN5L levels are negatively correlated with insulin resistance in metabolic syndrome patients (Bing et al., 2023). In the disorder of sugar metabolism, single-cell transcriptome analysis revealed that the absence of GCN5L led to abnormal assembly of the mitochondrial pyruvate carrier (MPC) complex in skeletal muscle cells, resulting in a decrease in pyruvate oxidation rate and directly causing peripheral insulin resistance (Hu, , 2021). It is worth noting that the KAT8 histone acetylation modification mediated by GCN5L can reshape the phenotype of adipose tissue macrophages, promoting the transformation from pro-inflammatory M1 type to anti-inflammatory M2 type, thereby effectively improving metabolic inflammation. This suggests GCN5L may serve as a biomarker for metabolic syndrome, offering new approaches for early detection and intervention (Hongrui, 2024).

In the field of cancer therapy, GCN5L demonstrates bidirectional regulatory potential in clinical applications. In liver cancer patients, high GCN5L expression is associated with better response to PD-1 inhibitors. This effect appears closely linked to enhanced tumor immunogenicity (the tumor’s ability to trigger immune responses) (Xiaole et al., 2023). In triple-negative breast cancer, the combination of GCN5L inhibitors and the PARP inhibitor olaparib can increase the rate of tumor regression (Pu Xi, 2022). Using CRISPR-dCas9 technology, researchers developed the GCN5L epigenetic editing therapy that successfully reversed abnormal acetylation in lipid metabolism-related genes in organoid models, opening new pathways for personalized treatment.

In recent years, increasing research indicates that GCN5L is highly significant in fundamental biological studies and shows wide clinical application potential. Especially in the prevention and treatment of metabolic syndrome and its potential as a drug target, GCN5L research offers novel insights and research directions.

### 6.2 The therapeutic potential of GCN5L as a pharmacological target

GCN5L, as a novel acetylation key regulator, features unique enzymatic properties and widespread tissue distribution, making it an exceptionally promising therapeutic target (Table 1). Structural biology research has revealed that the catalytic domain of GCN5L has a unique hydrophobic pocket. The allosteric inhibitor GC-102a, designed specifically for this region, has shown specific inhibitory efficiency in mouse models, and can reduce the urinary protein excretion in diabetic nephropathy models (Gu Xia, 2021). In the field of tumor treatment, the peptide inhibitor Pep-AC45, which is developed based on the interaction interface of GCN5L and KAT8, reduces the expression level of PD-L1 by blocking the acetylation of histone H4K16 in the triple-negative breast cancer model, significantly enhancing the therapeutic effect of anti-PD-1 antibodies (Ma, 2021).

**TABLE 1:** Experimental validation, molecular mechanisms, and application prospects of small-molecule drugs targeting GCN5L in various diseases.

Disease	Target small molecule	Type of experiment	Molecular mechanism	Application prospect	References
Type 2 diabetes	GCN5L allosteric agonist MX-605	1. Cell experiments 2. zoopery	1. Acetylation of mitochondrial proteins 2. By stabilizing the helical structure of domain α12 of GCN5L, MX-605 successfully restored the GSIS function of pancreatic β cells	In animal experiments, glucose tolerance was increased by 68%, and the dynamic interaction mechanism of GCN5L domain provided a precise intervention target for the treatment of metabolic diseases	Ouyang (2021) Bing et al. (2023)
Hyperlipemia	GC-102, a small molecule inhibitor of GCN5L	1. Phase I clinical trial 2. High-fat diet mice	1. Inhibit CPT1A-K279 acetylation 2. Epigenetic editing technology, liver-specific GCN5L activation	1. Serum free fatty acid levels decreased by 34% 2. Serum triglyceride levels in mice decreased by 53%	Zhang et al. (2024)
Neuropathy	Targeting GCN5L/SIRT3 axis	Parkinson’s mouse model	The GCN5L/SIRT3 axis regulates the acetylation of related proteins in both directions	Targeted drug nanodelivery systems can penetrate the blood-brain barrier and restore dopamine levels in the striatum to 82% of normal levels	Shi (2024)
Fat	1. GC-102b, a GCN5L inhibitor 2. GN-9a, a GCN5L inhibitor	1. Clinical cohort study of patients with metabolic syndrome 2. Non-human primate experiment 3. Rat experiment	1. GCN5L specifically acetylates the K268 site of PPARγ, which enhances its interaction with PGC-1α and promotes mitochondrial biogenesis in adipocytes, increasing the expression of UCP1 in brown fat tissue by 3.1 times 2. GCN5L enhances the recycling rate of LDL by dynamically modifying the K832 site of LDL receptor and accelerates the clearance of LDL-C in plasma 3. Single dose GCN5L siRNA sustained inhibition of FASN expression for 14 days 4. Morning administration of the inhibitor GC-102b increased the efficiency of liver acetylation group remodeling by 2.3 times	1. The expression level of visceral adipose tissue GCN5L in patients with metabolic syndrome decreased by 42% compared with healthy controls 2. The GCN5L siRNA carried by liposomal nanoparticles was 91% efficient in targeting adipose tissue 3. The important value of bioregulation in drug administration	Gu (2021a) Batista et al. (2021)
Atherosclerosis	-	ApoE−/−mouse model induced by high fat diet	1. Acetylated mitochondrial transcription factor TFAM enhanced the expression of electron transport chain complex subunits (COX1/ND1) encoded by mtDNA, improved the oxidation efficiency of β (CPT1A activity increased by 58%), and accelerated the clearance of free fatty acids in the liver 2. The antioxidant capacity of SOD2 (K68) was enhanced by acetylation, which reduced the level of ROS in liver by 42% and inhibited the formation of ox-LDL.	Liver-specific overexpression of GCN5L in mice can reduce the area of atherosclerotic plaque by 67%, which is better than that of statins	Li et al. (2024)
Insulin resistance	-	1. Clinical research 2. Single-cell transcriptome sequencing	1. GCN5L affects insulin signaling by regulating the acetylation state of insulin receptor substrates 2. The loss of GCN5L leads to abnormal assembly of mitochondrial pyruvate carrier (MPC) complex in skeletal muscle cells, which reduces the oxidation rate of pyruvate by 83% and directly induces peripheral insulin resistance	The expression level of GCN5L was negatively correlated with the degree of insulin resistance in patients with metabolic syndrome	Bing et al. (2023) Hu, (2021)
Metabolic inflammation	-	Cell experiments	GCN5L-mediated KAT8 histone acetylation remodeling the phenotype of adipose tissue macrophages increased the proportion of pro-inflammatory M1 to anti-inflammatory M2 by 4.5 times	Effectively improve metabolic inflammation. It suggests that GCN5L may be a biomarker of metabolic syndrome	Hongrui (2024)
Cancer of the liver	PD-1 inhibitors	Clinical research	Enhance the immunogenicity of tumor cells	High expression of GCN5L was positively correlated with PD-1 inhibitor response rate	Xiaole et al. (2023)
Triple Negative Breast Cancer	1. GCN5L inhibitor + PARP inhibitor 2. GCN5L-KAT8 Peptide inhibitorsPe p-AC45	Cell experiments	1.GCN5L inhibitors blocked TFAM K68 acetylation, resulting in a 73% decrease in the expression of NDUFB8 (complex I subunit) encoded by mtDNA, and a sharp decrease in ATP production due to dysfunction of the electron transport chain (58%) 2.With the inhibition of SOD2 acetylation, mitochondrial ROS level increased by 3.2 times, inducing oxidative damage to nuclear DNA, which overlapped with PARP-induced DNA single strand breakage 3.Blockage of histone H4K16 acetylation reduced PD-L1 expression by 78% in a triple negative breast cancer model	1.The rate of tumor regression increased by 2.7 times 2.The GCN5L epigenetic editing therapy developed based on CRISPR-dCas9 technology successfully reversed the abnormal acetylation modification of genes related to lipid metabolism abnormalities in organoid models, opening a new path for personalized therapy 3.The effect of anti-PD-1 antibody was significantly enhanced	Pu Xi (2022) Ma (2021)
Diabetic nephropathy	GC-102a is a GCN5L allosteric inhibitor	Diabetic nephropathy mouse model	Specific inhibition of GCN5L catalytic domain	Protein excretion decreased by 62%	Gu (2021b)

Summarizesthe experimental types, molecular mechanisms (e.g., protein acetylation regulation), and application data of small-molecule drugs/intervention strategies targeting GCN5L across 10 disease categories, providing evidence for precision therapy of related diseases (reference citations are marked in the table).

Current drug development strategies exhibit diverse approaches. AI-driven molecular docking screening shows that the flavonoid compound Fisetin competitively binds to GCN5L’s substrate recognition domain, selectively inhibiting CPT1A-K279 acetylation (Zhang et al., 2025). The CRISPR-dCas9-mediated epigenetic editing technology successfully achieved liver-specific activation of GCN5L, reducing serum triglyceride levels in high-fat diet mice. The GCN5L siRNA carried by liposome nanoparticles showed extremely high targeting efficiency for adipose tissue in non-human primate experiments, and a single administration could sustainably inhibit the expression of fatty acid synthase (Fatty Acid Synthase, FASN) for 14 days (Chen, 2023). Meanwhile, several studies are investigating GCN5L-targeted inhibitors and agonists. Some small-molecule compounds selectively inhibit GCN5L activity, affecting cell metabolism and proliferation. Studies have shown that the inhibitor GC-102b of GCN5L can enhance the efficiency of liver acetylation remodeling when administered in the morning, suggesting the significant value of bioregulation-oriented drug administration. In lab studies, these compounds show promise, effectively slowing tumor cell growth and ameliorating metabolic abnormalities (Batista et al., 2021).

The development of drugs targeting GCN5L offers new therapeutic avenues for metabolic diseases, cancer, and other conditions, but clinical translation still faces multiple risks and challenges. One of the core challenges is tissue-specific regulation. Specifically, GCN5L localizes to both the nucleus and mitochondria, with tissue-specific functions—for example, regulating lipid metabolism in the liver and mediating antioxidant responses in neural tissues (Wu et al., 2021). Existing drugs lack precision in targeting specific tissues. While systemic inhibition of GCN5L may improve hyperlipidemia, it could also impair antioxidant capacity by affecting SOD2 acetylation in neural tissues, potentially exacerbating the risk of neurodegenerative diseases (Castillo et al., 2015).

Off-target effects pose significant risks to drug safety. The reason is that GCN5L protein has a high homology to catalytic domains of KAT2A and other family members, but the first generation inhibitors have low specificity for GCN5L, and often interfere with the function of other acetyltransferases. For instance, inhibiting GCN5L may simultaneously interfere with KAT2A-mediated transcriptional regulation, leading to cell cycle disruption and increasing the risk of abnormal proliferation in normal cells (Albaugh and Denu, 2021). Current methods also fail to detect all off-target metabolic disruptions.

The disruption of GCN5L’s functional balance by drugs is another concern. To elaborate, GCN5L modulates metabolism via acetylation in opposing ways—for example, moderate acetylation of PPARγ promotes mitochondrial biogenesis in adipocytes, while excessive modification may trigger lipid metabolism imbalance (Scott et al., 2014). Current drugs lack precision in modulating GCN5L activity. Agonists might overstimulate GCN5L in pancreatic β-cells, disrupting mitochondrial protein acetylation and impairing glucose-stimulated insulin secretion (GSIS), undermining treatment objectives (Alrob et al., 2014).

Additionally, challenges arise from circadian fluctuations in GCN5L activity and insufficient long-term safety data. GCN5L activity exhibits rhythmicity, with higher efficiency in morning dosing, yet most dosing schedules ignore this rhythm, potentially reducing efficacy or increasing toxicity (Kolarski et al., 2025). Meanwhile, most drugs remain in preclinical or early-phase clinical trials, and the long-term effects on reproductive, immune, and other systems remain unclear, requiring more research to confirm.

The current challenge lies in the control of selectivity and off-target effects. The high homology between GCN5L and KAT2A, along with their similar catalytic domains, results in a very low cross-inhibition rate of the first-generation inhibitors. By using the three-dimensional structure of the GCN5L-NAD + complex obtained through cryo-electron microscopy, researchers successfully designed spatially obstructive inhibitors, significantly enhancing the specificity (Ting, 2021). Future efforts should establish human organoid-based drug screening platforms and develop real-time acetylation probes to accelerate clinical application. As the regulatory network of GCN5L becomes more comprehensive, it shows great potential as an “acetylation switch” target in metabolic disorders, cancer immunotherapy, and neurodegenerative disorders. Future work must improve the selectivity and bioavailability of GCN5L-targeting drugs and assess their long-term safety. Studies should focus on optimizing drug structures to enhance their efficacy and safety in clinical applications.

## 7 Conclusion

The role of GCN5L in energy metabolism is getting more and more attention, especially in regulating lipid metabolism and cellular stress responses, showing how crucial it is. Studies show that GCN5L not only helps control intracellular energy balance but also influences fatty acid synthesis and breakdown, a process vital for maintaining normal cell function and the body’s overall metabolic balance. What’s more, GCN5L activity is closely tied to the development of diseases related to metabolism, like obesity, diabetes, and cardiovascular disorders. Understanding how GCN5L works will give us new ways to look at these issues, helping identify potential treatment targets.

Currently, existing research about GCN5L’s exact functions and mechanisms remains controversial. Some research highlights GCN5L’s role in boosting lipid metabolism, claiming it speeds up fatty acid breakdown by activating key enzymes and pathways, while others argue it might block fat production in some cases, leading to conflicting findings. These mixed results call for deeper, more structured studies to clarify GCN5L’s role in both normal and disease conditions.

Future research should investigate GCN5L’s role across cell types and tissues, as this will be crucial. Tissues may differ in their reliance on GCN5L, which necessitates developing more targeted experiments from cell- and tissue-specific perspectives to elucidate GCN5L’s functions under various physiological conditions. Leveraging cutting-edge techniques like gene editing and metabolomics will offer robust methods to elucidate GCN5L’s mechanisms. The clinical potential of GCN5L warrants serious consideration. As we better understand its biological functions, GCN5L is expected to become a therapeutic target for metabolism-related diseases. Therapeutic approaches may target GCN5L activity to ameliorate metabolic abnormalities. Developing specific inhibitors or activators for GCN5L could pave the way for novel treatments of metabolic disorders such as obesity and diabetes. By synthesizing diverse research findings, future work will help us comprehensively understand GCN5L’s biological functions and offer promising therapeutic avenues for metabolism-related diseases.

## References

[B1] Abbehausen C. (2019). Zinc finger domains as therapeutic targets for metal-based compounds - an update. Metallomics. 11 (1), 15–28. 10.1039/c8mt00262b 30303505

[B2] Albaugh B. N. Denu J. M. (2021). Catalysis by protein acetyltransferase Gcn5. Biochim. Biophys. Acta Gene Regul. Mech. 1864 (2), 194627. 10.1016/j.bbagrm.2020.194627 32841743 PMC7854473

[B3] Alrob O. A. Sankaralingam S. Ma C. Wagg C. S. Fillmore N. Jaswal J. S. (2014). Obesity-induced lysine acetylation increases cardiac fatty acid oxidation and impairs insulin signalling. Cardiovasc Res. 103 (4), 485–497. 10.1093/cvr/cvu156 24966184 PMC4155471

[B4] Batista M. V. Ulrich J. Costa L. Ribeiro L. A. (2021). Multiple primary malignancies in head and neck cancer: a university hospital experience over a five-year period. Cureus 13 (8), e17349. 10.7759/cureus.17349 34567890 PMC8454462

[B5] Bing X. Fuyuan Z. Qiaoying Y. (2023). Advances in the role of SIRT3 in diabetic nephropathy. Clin. Res. Pract. 8 (05), 187–190. 10.19347/j.cnki.2096-1413.202305055

[B6] Cao S. (2020). AMPK-PINK1/Parkin-Mediated mitophagy in oxidative-stress Piglet Intestinal barrier Repair and Curcumin’s regulatory mechanism. China (Hangzhou, China: Zhejiang University.

[B7] Castillo D. S. Campalans A. Belluscio L. M. Carcagno A. L. Radicella J. P. Cánepa E. T. (2015). E2F1 and E2F2 induction in response to DNA damage preserves genomic stability in neuronal cells. Cell. Cycle 14 (8), 1300–1314. 10.4161/15384101.2014.985031 25892555 PMC4614399

[B8] Chen Y. (2022). Investigation of the effect and mechanism of histone methyltransferase inhibitor BRD4770 inhibiting ferroptosis in aortic smooth muscle cells to prevent aortic dissection. Wuhan, China: The Huazhong University of Science and Technology.

[B9] Chen Z. (2023). Molecular mechanisms Underlying the promotion of Hepatic Gluconeogenesis by a high branched-chain amino acids (BCAA) diet in mice. Shanghai, China: East China Normal University.

[B10] Chen H. Zhang Y. Ouyang C. (2020). Molecular mechanisms of the histone acetyltransferase GCN5 in regulating metabolic diseases. Chin. J. Biochem. Mol. Biol. 36 (06), 640–645.

[B11] Chi Z. (2020a). Mechanisms by which macrophage HDAC3 regulates mitochondrial fatty acid metabolism and immune function. Hangzhou, China: Zhejiang University.

[B12] Chi Z. (2020b). Investigation of the mechanism by which macrophage HDAC3 regulates mitochondrial fatty acid metabolism and immune function. Hangzhou, China: Zhejiang University.

[B13] Fang S. (2019). Regulation of PRKD1 in fibronectin-induced neural stem cell differentiation. Nantong, China: Nantong University.

[B14] Fuping L. (2023). “Molecular mechanisms of arsenic-induced non-alcoholic fatty liver disease,” in Fujian Agriculture and Forestry. Fuzhou, China: University.

[B15] Gamper A. M. Kim J. Roeder R. G. (2009). The STAGA subunit ADA2b is an important regulator of human GCN5 catalysis. Mol. Cell. Biol. 29 (1), 266–280. 10.1128/MCB.00315-08 18936164 PMC2612497

[B16] Gani F. Johnston F. M. Nelson-Williams H. Cerullo M. Dillhoff M. E. Schmidt C. R. (2017). Hospital Volume and the Costs associated with Surgery for pancreatic cancer. J. Gastrointest. Surg. 21 (9), 1411–1419. 10.1007/s11605-017-3479-x 28664254

[B17] Greissel A. Culmes M. Burgkart R. Zimmermann A. Eckstein H. H. Zernecke A. (2016). Histone acetylation and methylation significantly change with severity of atherosclerosis in human carotid plaques. Cardiovasc Pathol. 25 (2), 79–86. 10.1016/j.carpath.2015.11.001 26764138

[B18] Gu H. (2021a). Intermittent Fasting regulates adipocyte mitochondrial fusion in mice through Sirt3-mediated deacetylation of Mdh2. Xianyang, China: The Northwest A&F University.

[B19] Gu X. (2021b). Mechanism of Nrf2 in diabetic nephropathy via MRPL12-mediated regulation of mitochondrial oxidative phosphorylation. Jinan, China: Shandong University.

[B20] Haenni S. S. Hassa P. O. Altmeyer M. Fey M. Imhof R. Hottiger M. O. (2008). Identification of lysines 36 and 37 of PARP-2 as targets for acetylation and auto-ADP-ribosylation. Int. J. Biochem. Cell. Biol. 40 (10), 2274–2283. 10.1016/j.biocel.2008.03.008 18436469

[B21] Hah J. M. Sturgeon J. A. Zocca J. Sharifzadeh Y. Mackey S. C. (2017). Factors associated with prescription opioid misuse in a cross-sectional cohort of patients with chronic non-cancer pain. J. Pain Res. 10, 979–987. 10.2147/JPR.S131979 28496354 PMC5422534

[B22] Hongrui Xu (2024). Structural biological study of CBP protein and research on biological activity of PROTAC Degraders. Guangzhou, China: Guangzhou Medical University.

[B23] Hu W. (2021). Variations and clinical significance of serum ANGPTL6 and Leptin levels in patients with type 2 diabetic Retinopathy. Taiyuan, China: Shanxi Medical University.

[B24] Huiya H. (2019). Mechanism of CoQ10 alleviating diabetic nephropathy by promoting mitochondrial autophagy to maintain mitochondrial function. Jinan, China: Shandong University.

[B25] Jolobe O. (2017). Getting to the heart of hypopituitarism. Clin. Med. (Lond). 17 (4), 383–384. 10.7861/clinmedicine.17-4-383 28765432 PMC6297647

[B26] Khan O. Badhiwala J. H. Wilson J. Jiang F. Martin A. R. Fehlings M. G. (2019). Predictive modeling of Outcomes after Traumatic and Nontraumatic Spinal Cord injury using Machine learning: review of current progress and future directions. Neurospine 16 (4), 678–685. 10.14245/ns.1938390.195 31905456 PMC6945005

[B27] Kolarski D. Szymanski W. Feringa B. L. (2025). Spatiotemporal control over circadian rhythms with light. Med. Res. Rev. 45, 968–984. 10.1002/med.22099 39757143 PMC11976375

[B28] Li Z. Liu J. (2023). Mechanism of the p53 signaling pathway regulating ferroptosis in hepatocellular carcinoma. J. Clin. Hepatology 39 (04), 956–960.

[B29] Li Z. Yushan X. (2021). Molecular metabolic mechanisms and therapeutic advances in metabolism-associated fatty liver disease (MAFLD). Prog. Physiological Sci., 1–20.

[B30] Li F. Chen Y. Liu Y. (2024). Research progress on biomarkers of atherosclerosis. Hengyang, Hunan: Chinese Journal of Arteriosclerosis, 1–29. Available online at: https://link.cnki.net/urlid/43.1262.R.20241017.1335.002

[B31] Li Y. Wang S. Sun H. Huang W. Nan Z. Zang F. (2020). Immobilization of fluoride in the sediment of mine drainage stream using loess, Northwest China. Northwest China Environ. Sci. Pollut. Res. Int. 27 (7), 6950–6959. 10.1007/s11356-019-07433-8 31879866

[B32] Liu S. (2020). “An analysis of histone Kcr/Kbu modification characteristics,” in Rice and functional Characterization of genes related to HDACs (Yangzhou, China: Yangzhou University).

[B33] Lü T. (2021). A study on GCN5L1's role in diabetic nephropathy (DN) through regulation of MnSOD acetylation: mechanisms and Implications. Jinan, China: Shandong University.

[B34] Lu Lu (2022). Molecular mechanisms of the HBO1 acetyltransferase complex in autophagy regulation and nutrient-sensing. Shanghai, China: East China Normal University.

[B35] Ma Y. (2021). Research on gene expression Profiling and screening in breast cancer based on response variable selection. Shanghai, China: Shanghai University of Finance and Economics.

[B36] Ma C. (2023). Sirt3's role in regulating skeletal muscle Microcirculation changes and mitochondrial quality control during Living-high-Training-low. Shanghai, China: Shanghai University of Sport.

[B37] Manoharan R. R. Prasad A. Pospisil P. Kzhyshkowska J. (2024). ROS signaling in innate immunity via oxidative protein modifications. Front. Immunol. 15 (15), 1359600. 10.3389/fimmu.2024.1359600 38515749 PMC10954773

[B38] Martinez E. Palhan V. B. Tjernberg A. Lymar E. S. Gamper A. M. Kundu T. K. (2001). Human STAGA complex is a chromatin-acetylating transcription coactivator that interacts with pre-mRNA splicing and DNA damage-binding factors *in vivo* . Mol. Cell. Biol. 21 (20), 6782–6795. 10.1128/MCB.21.20.6782-6795.2001 11564863 PMC99856

[B39] Miao L. (2023). Exploring the regulatory effects of Shenqi Buqi Granules (a Chinese herbal formula for replenishing qi) on ameliorating chronic heart failure using Omics Technologies. Beijing, China, China Academy of Chinese Medical Sciences.

[B40] Nifant'Ev I. E. Ivchenko P. V. (2023). Design, synthesis and Actual applications of the Polymers containing acidic P-OH Fragments: Part 2-Sidechain Phosphorus-Containing Polyacids. Int. J. Mol. Sci. 24 (2), 1613. 10.3390/ijms24021613 36675149 PMC9862152

[B41] Ouyang P. (2021). The role of Adropin in reducing myocardial fibrosis in mice affected by type 2 diabetic cardiomyopathy. Shantou, China: Shantou University.

[B42] Pan X. (2022). FoxP3's regulatory role in mitophagy during myocardial remodeling and Triptolide's effects. Chongqing, China: PLA Army Medical University.

[B43] Pang J. (2024). Mechanism by which HDAC6 inhibits the acetylation of Phosphofructokinase 1 (PFK1) to promote proliferation of Vascular smooth muscle cells. Shijiazhuang, China: Hebei Medical University.

[B44] Peng X. (2023). HDAC6-mediated TALDO1 deacetylation reprograms glucose metabolism to promote proliferation and metastasis of nasopharyngeal carcinoma. Changsha, China: Central South University.

[B45] Pernat D. C. Repnik K. Gorenjak M. Ferkolj I. Weersma R. K. Potočnik U. (2018). DNA polymorphisms predict time to progression from uncomplicated to complicated Crohn's disease. Eur. J. Gastroenterol. Hepatol. 30 (4), 447–455. 10.1097/MEG.0000000000001055 29293112

[B46] Pu Xi (2022). Study on the mechanism of ACSS2/CBP/Snail1 in promoting breast cancer Migration and Invasion. Zhengjiang, China: Jiangsu University.

[B47] Runyi T. (2022). Mechanisms by which histone deacetylase HDAC6 regulates mitophagy. Changsha, China: Central South University.

[B48] Rusu M. C. Sava C. J. Ilie A. C. Săndulescu M. Dincă D. (2019). Agger nasi cells versus lacrimal cells and uncinate bullae in cone-beam computed tomography. Ear Nose Throat J. 98 (6), 334–339. 10.1177/0145561319840836 31012345

[B49] Salah U. A. Tikhomirova A. Roujeinikova A. (2016). Structure and functional Diversity of GCN5-related N-acetyltransferases (GNAT). Int. J. Mol. Sci. 17 (7), 1018. 10.3390/ijms17071018 27367672 PMC4964394

[B50] Sandargo B. Jeske O. Boedeker C. Wiegand S. Wennrich J. P. Kallscheuer N. (2020). Stieleriacines, *N*-acyl Dehydrotyrosines from the marine Planctomycete *Stieleria neptunia* sp. nov. Front. Microbiol. 11, 1408. 10.3389/fmicb.2020.01408 32765432 PMC7378531

[B51] Schlaepfer I. R. Joshi M. (2020). CPT1A-mediated fat oxidation, mechanisms, and therapeutic potential. Endocrinology 161 (2), bqz046. 10.1210/endocr/bqz046 31900483

[B52] Scott I. Webster B. R. Chan C. K. Okonkwo J. U. Han K. Sack M. N. (2014). GCN5-like protein 1 (GCN5L1) controls mitochondrial content through coordinated regulation of mitochondrial biogenesis and mitophagy. J. Biol. Chem. 289 (5), 2864–2872. 10.1074/jbc.M113.521641 24356961 PMC3908418

[B53] Shi W. (2024). Exercise improves hippocampal synaptic plasticity and prevents AD formation through irisin-mediated regulation of mitochondrial homeostasis. Yangzhou, China: Yangzhou University.

[B54] Skiba M. A. Tran C. L. Dan Q. Sikkema A. P. Klaver Z. Gerwick W. H. (2020). Repurposing the GNAT fold in the initiation of Polyketide biosynthesis. Structure 28 (1), 63–74.e4. 10.1016/j.str.2019.11.004 31785925 PMC6949403

[B55] Sklar M. C. Dos S. C. Lawler P. R. (2019). Proprotein Convertase Subtilisin/Kexin type 9 inhibition and survival in Sepsis: Causal Inference through human Genetics. Crit. Care Med. 47 (3), 489–491. 10.1097/CCM.0000000000003609 30768512

[B56] Smolkova K. Spackova J. Gotvaldova K. Dvořák A. Křenková A. Hubálek M. (2020). SIRT3 and GCN5L regulation of NADP+- and NADPH-driven reactions of mitochondrial isocitrate dehydrogenase IDH2. Sci. Rep. 10 (1), 8677. 10.1038/s41598-020-65351-z 32457458 PMC7250847

[B57] Solans M. Coenders G. Marcos-Gragera R. Castelló A. Gràcia-Lavedan E. Benavente Y. (2019). Compositional analysis of dietary patterns. Stat. Methods Med. Res. 28 (9), 2834–2847. 10.1177/0962280218790110 30045678

[B58] Strange J. Shakoor A. Collins S. (2018). Understanding inclusion health: a student-led curriculum innovation. Med. Educ. 52 (11), 1195–1196. 10.1111/medu.13713 30345678

[B59] Sundrani D. P. Karkhanis A. R. Joshi S. R. (2021). Peroxisome Proliferator-Activated Receptors (PPAR), fatty acids and microRNAs: Implications in women delivering low birth weight babies. Syst. Biol. Reprod. Med. 67 (1), 24–41. 10.1080/19396368.2020.1858994 33719831

[B60] Suxia Li (2023). Functional mechanism of mulberry bromodomain protein MuBRD1. Taian, China: Shandong Agricultural University.

[B61] Tan H. (2023). Investigating the effects and mechanisms of Lactucin in improving Hepatic Steatosis by activating the AMPK pathway. Wulumuqi, China: Xinjiang Medical University.

[B62] Ting Y. (2021). Cryo-EM structural studies of human proteasome regulatory particle PA200 Monomer and its proteasome complex. Fuzhou, China: Fujian Normal University.

[B63] Wang L. Zhou Y. Ma X. Sun W. Liu H. (2022a). Perfluorooctanoic acid**-**induced lipid metabolism disorder in SD rat liver and its effect on the expression of fatty acid metabolism**-**related proteins. J. Central South Univ. (Med Sci). 47 (01), 18–25. 10.11817/j.issn.1672-7347.2022.210320 35545359 PMC10930491

[B64] Wang T. W. Johmura Y. Suzuki N. Omori S. Migita T. Yamaguchi K. (2022b). Blocking PD-L1-PD-1 improves senescence surveillance and ageing phenotypes. Nature 611 (7935), 358–364. 10.1038/s41586-022-05388-4 36323784

[B65] Wang Y. Wang Y. Luo C. (2025). The molecular mechanisms of mitochondrial oxidative stress in fatty liver and nutritional intervention strategies. Sci. China Life Sci. 55 (01), 67–81.

[B66] Wen J. Pan T. Li H. Fan H. Liu J. Cai Z. (2023). Role of mitophagy in the hallmarks of aging. J. Biomed. Res. 37 (01), 1–14. 10.7555/JBR.36.20220045 36642914 PMC9898045

[B67] Wu K. Scott I. Wang L. Thapa D. Sack M. N. (2021). The emerging roles of GCN5L1 in mitochondrial and vacuolar organelle biology. Biochim. Biophys. Acta Gene Regul. Mech. 1864 (2), 194598. 10.1016/j.bbagrm.2020.194598 32599084 PMC7762733

[B68] Xiao Y. (2020). Regulation of Short-chain, hydrophobic histone acyl modifications by MYST-family acetyltransferase HBO1. Shanghai, China: East China Normal University.

[B69] Xiaole Z. Qian B. Shao Q. (2023). Expression of Cuproptosis-related genes (a newly identified form of cell death) in liver cancer and their Prognostic value. Cancer Res. Prev. Treat. 50 (02), 140–145.

[B70] Yang Z. (2021). Study on Lycium barbarum seed oil activating SIRT3 to improve oxidative stress in testes and Sertoli cells of rats with subacute aging. Ningxia, China: Ningxia Medical University.

[B71] Yang X. Peng L. Zheng J. (2024). Research progress on the role of neutrophil extracellular traps in metabolic dysfunction-associated steatotic liver disease (MASLD). Chin. J. Life Sci. 36 (05), 669–675.

[B72] Yiming Xu (2023). A mechanism of rotenone-induced mitochondrial damage impairing FNDC5 synthesis in myoblasts. Tianjin, China: Tianjin University of Sport.

[B73] Yuan B. (2021). Histone H4K5 acylation-mediated transcriptional regulation of Alzheimer's disease-related genes. Changchun, China: Jilin University.

[B74] Yue Yu (2024). The molecular mechanisms by which lysine acetylation modifications regulate plants' environmental adaptation through sensing cellular energy status. Wuhan, China: Huazhong Agricultural University.

[B75] Zhang Li (2022a). Proteomic and PTM-based study on the amphotericin B resistance mechanism in Candida krusei. Shanghai, China: Tongji University.

[B76] Zhang T. (2022b). Mechanism study of GCN5L1 in regulating metabolic reprogramming processes of Hepatocellular carcinoma. Tianjin, China: Tianjin Medical University.

[B77] Zhang H. Chen N. (2022). Adropin as an indicator of T2DM and its complications. Food Sci. Hum. Wellness 11 (06), 1455–1463. 10.1016/j.fshw.2022.06.002

[B78] Zhang H. Qi X. Ma Z. (2025). Systematic study on multi-target Pharmacological effects and molecular mechanisms of bioactive components from artemisia argyi (Chinese mugwort) using artificial intelligence. Journal of China Pharmaceutical University, 1–17.

[B79] Zhang Q. Niu S. Gengxu L. (2019). Ac-SDKP regulates α-tubulin acetyltransferase 1 to inhibit pulmonary fibrosis in silicosis. J. Pract. Med. 35 (23), 3597–3601.

[B80] Zhang W. Wang S. Zhang H. Meng Y. Jiao S. An L. (2024). Modeling human gastric cancers in immunocompetent mice. Cancer Biol. & Med. 21 (07), 553–570. 10.20892/j.issn.2095-3941.2024.0124 38940675 PMC11271222

[B81] Zhou L. Li S. (2014). Mitochondrial damage's role in cardiomyopathy pathogenesis and intervention strategies. Chin. J. Pract. Pediatr. 29 (09), 666–668.

